# Unit managers between fluctuating demand and fixed staffing: a quantitative study in psychiatric nursing

**DOI:** 10.3389/frhs.2026.1751261

**Published:** 2026-01-30

**Authors:** Michael Ketzer, Beatrice Gehri, André Nienaber, Christian G. Huber, Michael Simon

**Affiliations:** 1 Institute of Nursing Science, Department Public Health, University of Basel, Basel, Switzerland; 2 University Psychiatric Clinics Basel, University of Basel, Basel, Switzerland; 3 Faculty of Business Management and Social Sciences, Osnabrück University of Applied Sciences, Osnabrück, Germany

**Keywords:** bed occupancy, health workforce, hospital bed capacity, inpatients, leadership, nurses, personnel staffing and scheduling, psychiatric nursing

## Abstract

Balancing patient demand with nurse staffing remains a central challenge in inpatient care. In psychiatric settings, patient-side fluctuations create variability that is difficult to reconcile with fixed rosters and limited staffing flexibility. This study quantifies temporal variations in unit capacity utilization in psychiatric inpatient care and explores how unit managers respond to fluctuations and perceive flexible working arrangements. We combined routine inpatient data with a survey of unit managers from Swiss psychiatric hospitals. Routine data were used to describe temporal variability in capacity utilization, while the survey assessed management strategies, causes of workload fluctuations, and attitudes toward flexible working arrangements. Routine data from 116 units across 13 hospitals revealed substantial temporal fluctuations in occupancy. Most unit managers reported maintaining planned staffing levels despite changing demand, relying primarily on individual nurse-level adjustments such as overtime or calling in off-duty staff. Patient-side or structural strategies, including transfers or bed closures, were rarely used. Flexible working arrangements were viewed positively for nurse retention but deemed difficult to implement within shift-based operations. Psychiatric inpatient care illustrates the challenge of aligning fluctuating demand with staffing systems designed for stability. Current responses rely mainly on reactive measures that strain staff and may affect treatment continuity and safety, while opportunities for structural flexibility remain underused. Future research should develop data-driven tools to anticipate workload peaks and evaluate interventions that support flexible staffing and staff well-being. Organizational and policy efforts are needed to strengthen nurse manager capacity, improve working conditions, and support workforce planning that safeguards patient care.

## 1 Introduction

Balancing patient demand with available nurse staffing is a central challenge in inpatient care. Admissions, discharges, and changes in patient acuity create variability that must be managed within systems built on forward-planned staffing schedules, contractual hours, and institutional regulations. Although staffing is often planned to meet average demand, this approach inadequately reflects the dynamic nature of patient care [1]. Fluctuations can occur daily and even hourly, leading to temporary imbalances between workload and staffing [2].

Unit managers must navigate this variability while ensuring safe care for patients, maintaining the continuity of hospital processes, and protecting staff well-being. Predictable and reliable schedules are crucial for nurses’ health, performance, and job satisfaction [3]. In psychiatric settings, increasing nurses’ influence and predictability regarding their schedules has been linked to lower emotional exhaustion, reduced overtime, and stronger retention [4]. Balancing this desire for control with the flexibility demanded by patient care remains a core challenge in workforce planning.

Flexible staffing models-such as float pools, temporary staff, or flexible scheduling-have emerged as possible solutions. However, these approaches can introduce cost and coordination challenges [5]. Analytical studies propose stochastic or optimization-based approaches to solve nonstationary demand, but many rely on simplified assumptions that limit their practical relevance [6] and often overlook the well being of staff-a critical omission given the importance of retaining healthcare workers. Empirical evidence on how managers in psychiatric inpatient care respond to these fluctuations and perceive the feasibility of flexible arrangements is scarce.

This study addresses this gap in Swiss psychiatric inpatient care. Its aims are: (1) to quantify the magnitude of demand fluctuations and capacity utilization using routine data; (2) to examine how unit managers report responding to fluctuations; and (3) to explore their views on flexible working arrangements as potential responses.

## 2 Materials and methods

This study is part of the “Matching registered nurse services with changing care demands in psychiatric hospitals” (Match${}^{RN}$ Psychiatry) project, a multi-centre study in psychiatric hospitals in the German-speaking part of Switzerland [7]. The project combines cross-sectional nursing staff and unit surveys with routinely collected patient data to measure and describe the nursing work environment, staffing, and quality in psychiatric inpatient care.

All psychiatric hospitals represented in the Swiss Psychiatric Nursing Leaders’ Association (VPPS) (*n* = 40) were invited to participate. In 2023, 13 hospitals with 124 care units took part. Units were eligible if they (1) provided 24/7 inpatient care for adults and (2) were classified as non-forensic. Participating hospitals included university, public, and private institutions with inpatient capacities ranging from approximately 70 to 360 beds.

### 2.1 Data sources

Two data sources were used: (1) routine patient data extracted from clinical information systems and (2) an online unit manager survey. In each hospital, one designated contact person served as liaison for the study team and facilitated both data extraction and survey distribution, either by performing these tasks directly or by connecting the study team with the responsible internal staff.

#### 2.1.1 Patient routine data

Patient data were extracted for all discharges between 1 January and 31 December 2022 from 13 hospitals. These data were derived from four mandatory reporting files required by the Swiss national quality measurement programme [8] and were supplemented with the patient’s last inpatient unit to enable unit-level attribution. For the present analysis, we used only variables contained in the minimal dataset sheet (“MB dataset”), as defined by the Swiss Federal Statistical Office [9]. Variables included admission and discharge timestamps as well as patient demographic and clinical characteristics. All data were pseudonymised and securely transferred according to university-approved data protection standards.

Due to national reporting conventions, cases admitted within the reporting year but discharged thereafter are not available in the dataset and appear only in the subsequent year’s file. To ensure complete and comparable data across units, we therefore restricted the analysis to the nine months between January and September *2022*.

Unit size was derived from patient data by linking each patient’s stay to their assigned unit based on admission and discharge timestamps, generating a continuous occupancy profile for 2022. Unit size was defined as the maximum number of concurrent patients sustained for at least 1% of all hourly intervals. This data-based definition was used instead of self-reported size from the unit manager survey to minimize inconsistencies caused by differing interpretations of temporary or overflow beds.

Capacity utilization was calculated for each hour within the nine-month observation period as the ratio of occupancy to unit size. Daily averages were then computed as mean of the 24 hourly values. To visualize utilization patterns, a heatmap was generated. Because utilization data were right-skewed (with high values being more frequent), values were categorized to ensure approximately uniform cell densities across the color scale.

#### 2.1.2 Unit manager survey

The survey, conducted between November 2023 and April 2024, targeted unit managers or their deputies and collected information on staffing, service provision, and unit characteristics. Invitations were sent by email via the online survey system; email addresses of eligible respondents were provided by the designated hospital contact persons and managed within the survey platform for the purpose of survey administration. It comprised Likert-type items and additional open-ended text fields. Topics included imbalances between patient care demand and nursing staff supply, perceived causes, managerial responses, frequency of temporary bed closures, and reasons or barriers for implementing flexible work arrangements for nurses. The survey also collected demographic information on respondents’ education, work experience, and role (Supplementary File S1). Data were summarized using descriptive statistics and graphical representations.

### 2.2 Data analysis

All analyses were performed in R version 4.5.1 for MacOS. Descriptive statistics and graphical representations were produced using the tidyverse, likert and cowplot packages [10–13].

### 2.3 Ethical considerations

The study region’s responsible ethics commission (Ethics Commission Northwest and Central Switzerland) ruled that the Match^RN^ Psychiatry study is exempt from the Swiss Human Research Act (project ID: Req-2019-00589).

## 3 Results

**Figure 1:**
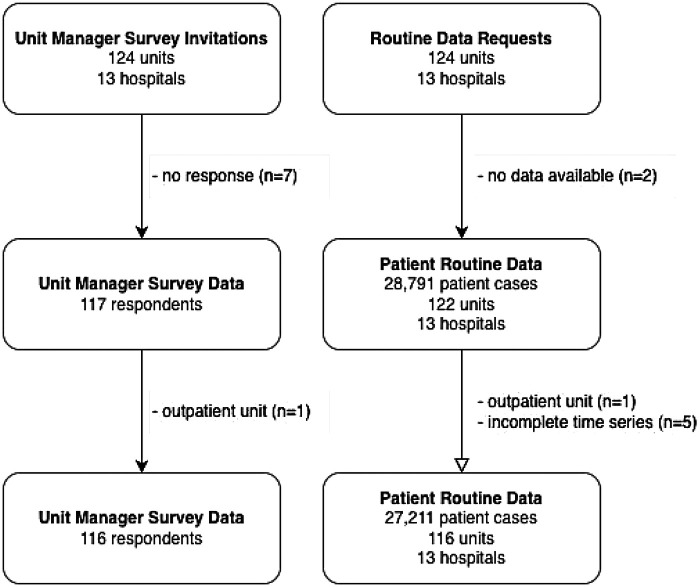
Data flow and final sample size.

**Figure 2:**
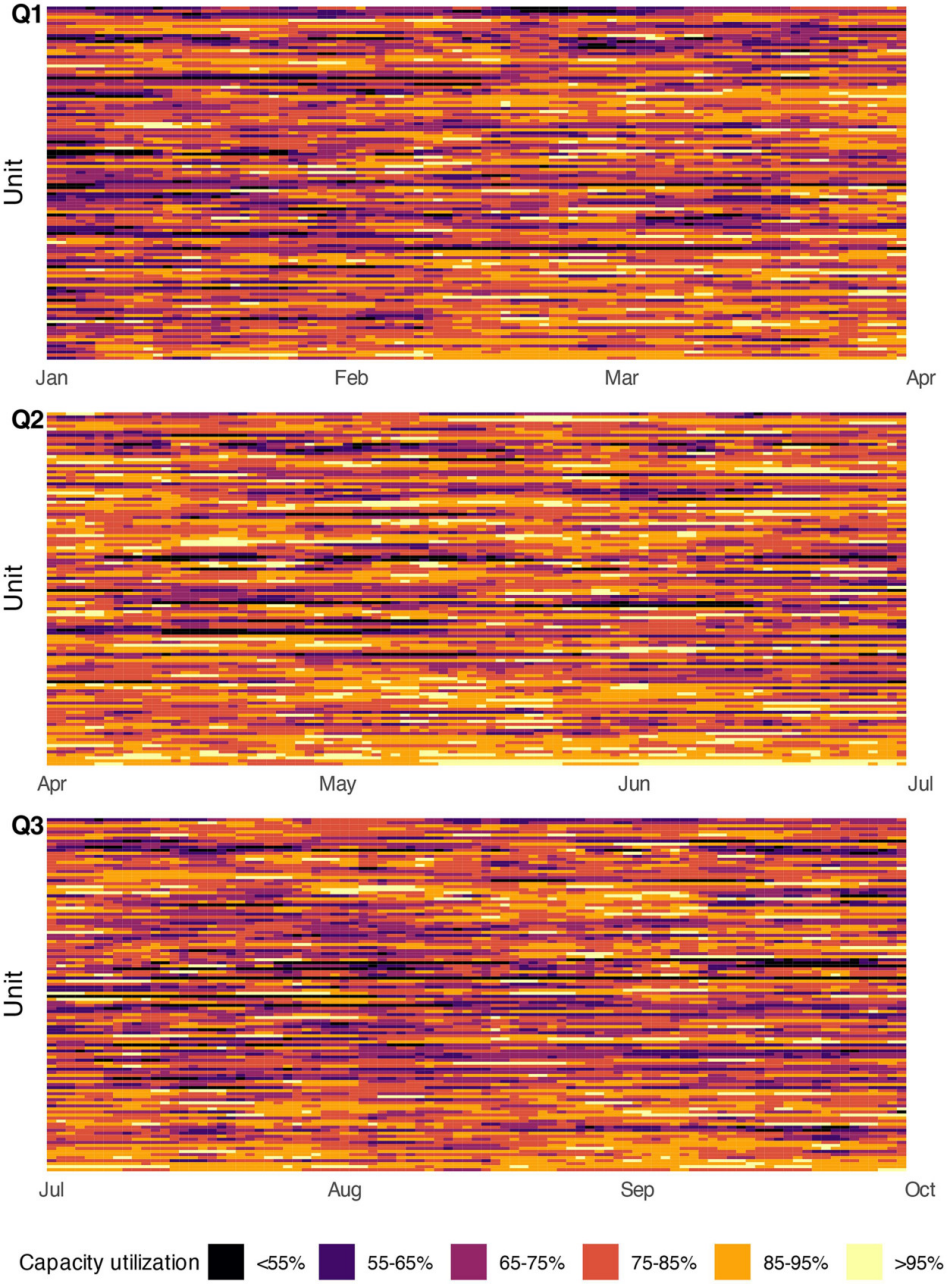
Daily capacity utilization of 116 inpatient units split in three quarters.

Addressing Aim 1, routine data from 116 units from 13 hospitals were analyzed; units with incomplete data, unclear temporal trends possibly suggesting unit size changes, or missing data were excluded (Figure 1). Median unit size was 21 (range 11–43) and mean daily capacity utilization was 78.8% (standard deviation 12%, range 62.7%–91.4%). Capacity utilization was divided into six categories: four 10% intervals from 55% to 95%, and one category each for values below 55% and above 95%. The heatmap (Figure 2) shows marked temporal variation in utilization across most units.

To address aims 2 and 3, data from the unit manager survey were analyzed. The response rate was 94%, (116 responses). Most participants were leaders of one or several units (*N* = 110), while three respondents were deputy unit managers (3 non-responses). Median duration of holding a leadership position was 5 years (IQR 3-10, range 0–35), median duration of working in the institution was 10 years (IQR 6-20, range 1–40), and median duration of working in nursing was 20 years (IQR 13-29, range 6–45). 19% (*N* = 22) of respondents hold a university degree (bachelor or master).

**Figure 3:**
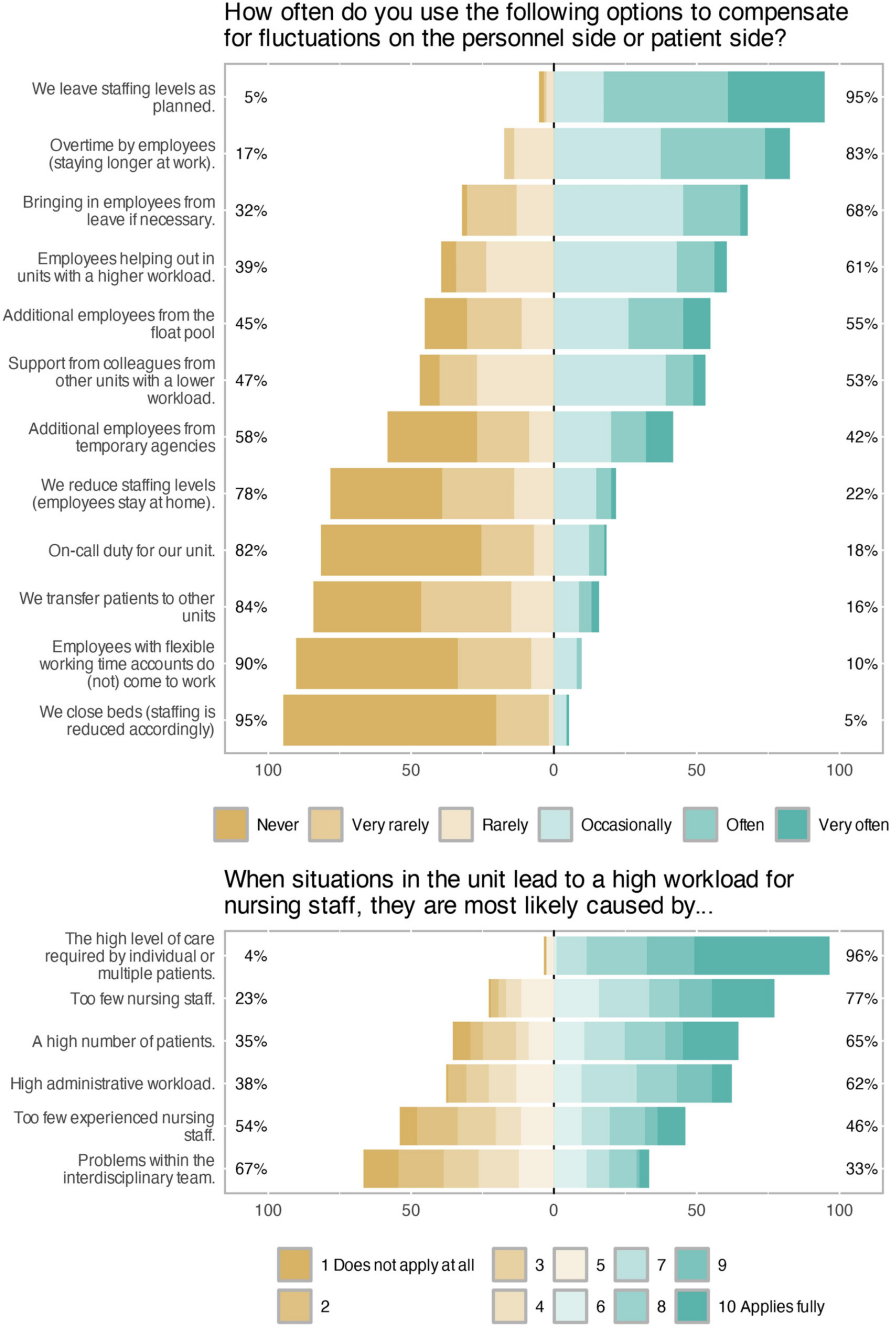
Unit managers’ responses to and perceived causes of fluctuations in demand.

With regard to aim 2, unit managers were asked how their units typically respond to fluctuations in patient care demand on the personnel or patient side. This question included six Likert-scale response options ranging from *never* to *very often*, see Figure 3 for full results. The predominant strategy was to maintain staffing levels as planned, meaning that no active adjustment is made to address imbalances between care needs and available nursing staff. This approach was reported as being used *very often*, *often*, or *occasionally* by 95% of respondents. The second most frequently reported measure was overtime by nurses (83%), followed by calling in staff who were not originally scheduled to work that shift (68%). Rarely applied strategies included transferring patients to other units (16%), deploying staff with flexible working time accounts (10%), and temporarily closing beds (5%). In free-text comments, two unit managers noted that they themselves occasionally step in to provide direct nursing care.

The closing of beds was also addressed in a separate survey question (“How often are beds closed in your unit (e.g., due to staff shortages)?”), to which 82.5% of unit managers reported that this *never* occurs, another 15.8% indicated closures *1–4 times per year*, while the remaining response options (*5–10 times per year*, *once a month*, *several times a month*, or *several times a week*) together accounted for 1.3% of responses.

When asked about the perceived causes of high nursing workload on their unit, managers rated several potential factors on a numeric scale from 1 (*does not apply at all*) to 10 (*applies fully*). The full distribution of ratings is shown in Figure 3. The factor most frequently identified as applying fully was a high level of care required by individual or multiple patients (96%), followed by an insufficient number of nursing staff (77%) and a high number of patients (65%). The least frequently endorsed cause was problems within the interdisciplinary team (33%). For descriptive purposes, responses from 1 to 5 were classified as *does not apply*, and responses from 6 to 10 as *does apply*. Free-text comments highlighted aggression or violence from patients (three mentions) and simultaneous admissions (two mentions) as additional causes of high workload. Single comments referred to language barriers, somatic comorbidities, and suicidality.

**Figure 4:**
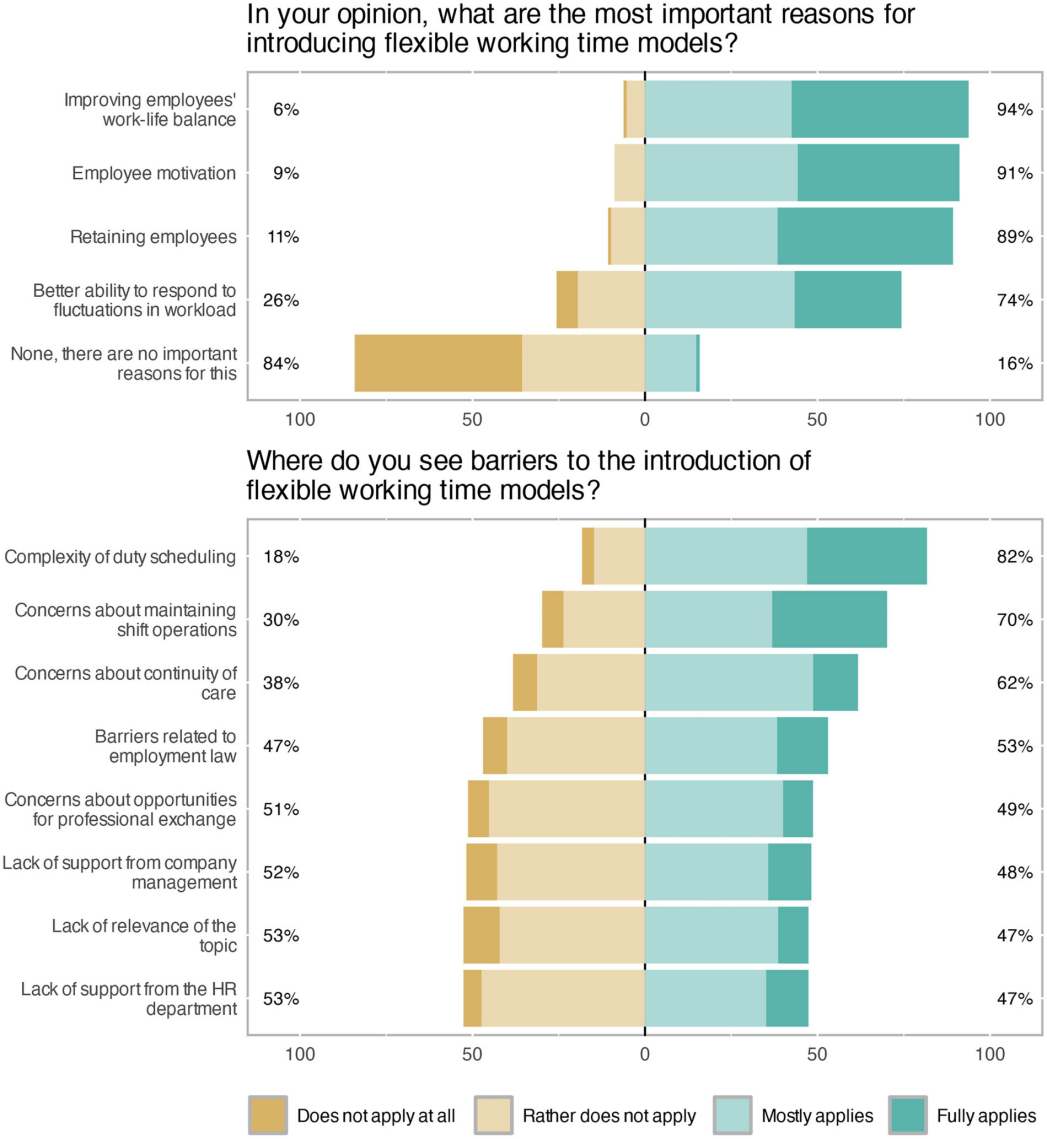
Reasons and barriers for flexible working arrangements.

Concerning aim 3, unit managers were asked about their attitudes toward and perceived barriers to implementing flexible working arrangements for nurses. Both questions used a four-point Likert scale ranging from *does not apply at all* to *fully applies*. The most frequently endorsed reasons in favor of flexible working arrangements were improving employees’ work–life balance (94%), enhancing motivation (91%), and retaining staff (89%). In free-text comments, the compatibility of work and family life was specifically mentioned three times. Overall, the responses show that most unit managers view flexible working time models as important for supporting and retaining nursing personnel. Detailed results are presented in Figure 4.

Perceived barriers to introducing flexible working arrangements are shown in Figure 4. The statements most frequently rated as applicable were the complexity of duty scheduling (82%), maintaining shift operations (70%), and ensuring continuity of care (62%). For all other potential barriers, including lack of support from management or human resources, and employment law constraints, responses were more evenly distributed, showing no clear majority either way.

## 4 Discussion

Using routine data and survey responses from unit managers, this study examined temporal variations in capacity utilization and strategies for managing fluctuations in psychiatric inpatient care. The results revealed substantial variability over time and showed that unit managers most commonly responded by maintaining planned staffing levels, meaning that short-term imbalances between care needs and available staff were rarely corrected. When adjustments were made, they occurred primarily on the nursing side-through overtime or by calling in off-duty staff-whereas patient-side measures, such as transfers or temporary bed closures, were seldom used. High workload was perceived mainly as patient-related, particularly due to high care intensity. Flexible working arrangements were viewed favorably for enhancing work-life balance, motivation, and retention, yet their implementation was perceived as constrained by the complexity of shift planning and the need to maintain shift operations and continuity of care.

These findings highlight a structural tension between the sources of workload fluctuations and the strategies used to manage them. Variability arises largely from patient-side dynamics (e.g., high level of required care) yet responses are sought almost exclusively on the staff side, often through reactive and short-term measures. This indicates that flexibility in psychiatric inpatient care currently takes place at an operational and primarily individual employee level (e.g., overtime, calling in staff) rather than a structural or strategic one (e.g., adaptive staffing models or resource pooling). Interestingly, barriers to flexible working arrangements are mostly perceived also on an operative level (e.g., complexity of duty scheduling), while structural or strategic barriers (e.g., employment law) are in comparison perceived to be less important. Together, these patterns indicate potential scope for more strategic approaches to managing fluctuations beyond the immediate operational responses. In this regard, staffing models that combine a fixed core workforce with a variable, demand-responsive component, already applied in parts of healthcare [14] and other sectors, could offer a promising approach, provided they are adapted to the specific requirements of psychiatric inpatient care and grounded in a sufficiently robust baseline staffing level to ensure safe care [15]. Implementing such approaches, however, depends on administrative decisions regarding contractual arrangements and resource allocation, which largely lie beyond the control of unit managers and require coordination and decision-making at a broader organizational level than the individual unit.

While flexible working arrangements are widely recognized as beneficial, the concept of “flexibility” may carry different meanings. For nurses, it typically refers to greater autonomy, predictability, and control over schedules; for managers, it implies responsiveness to fluctuating service demands. Greater perceived control and schedule autonomy, for example through self-scheduling, have been associated with improved well-being and job satisfaction [4, 16, 17]. However, implementing such models is challenging in shift-based work, where operational constraints limit managerial flexibility compared to non-clinical settings [18]. Moreover, effects on turnover, quality, and health outcomes remain context-dependent [19]. In nursing, flexible working arrangements often function less as tools for immediate staffing adjustments and more as mechanisms to align organizational practices with nurses’ need for stability and empowerment.

According to the unit manager survey, the three most frequently used strategies to compensate for fluctuations were maintaining planned staffing levels, requiring overtime, and calling in nurses on days off. Each of these approaches places additional strain on the nursing workforce employed on that unit. Maintaining planned staffing despite rising demand increases workload for those on shift. Overtime reduces opportunities for rest and work-life balance and has been linked to turnover intentions among nurses and to adverse events [20–22]. Similarly, calling in nurses on their days off is linked to fatigue and may undermine recovery between shifts [23]. In combination, these reactive operational measures may not only strain staff but may also jeopardize safe patient care when sustained over time.

In our survey, only a small minority of unit managers reported using patient-side measures such as transferring patients to other units when demand exceeded capacity. This pattern is consistent with the clinical drawbacks of transfers in psychiatric inpatient care, where interruptions to therapeutic continuity may prolong treatment duration and contribute to poorer outcomes [24]. Instead, more than half of the unit managers indicated that support was provided through cross-unit staffing, for example by sending nurses to other units in need of assistance or by having colleagues with available capacity come to their unit. Such approaches avoid the disruptions associated with patient transfers and may help maintain continuity, yet they also introduce coordination challenges. Moreover, cross-unit staffing requires nurses to work in unfamiliar environments, which necessitates sufficient induction or preparatory training to ensure safe care outside their usual clinical context. Given these demands, such assignments should ideally occur on a voluntary basis or be accompanied by appropriate compensation to acknowledge the additional flexibility expected of individual nurses. Temporary bed closures are also rarely used, likely because they entail substantial financial consequences for hospitals, as closing beds leads directly to revenue losses.

Temporary or agency nurses have attracted considerable public attention in Switzerland, largely due to concerns about their perceived high costs. The association of Zurich hospitals recently attempted, unsuccessfully, to restrict their deployment [25]. While this debate illustrates one aspect of the complexity of workforce planning, it represents only a small part of the broader challenges that managers face in maintaining adequate staffing and care quality. In our study, more than half of the unit managers reported employing temporary nurses never or only rarely, consistent with evidence that such measures alone do not effectively address staffing imbalances [26]. Safety concerns persist as well: higher reliance on agency or overtime staff has been linked to more pressure ulcers [27], and temporary staff only partly mitigate the increased mortality risk linked to low nurse staffing [28]. In psychiatric inpatient care, the use of temporary staff may be particularly problematic because therapeutic relationships, continuity, and detailed knowledge of patients’ histories are central to effective and safe care. The public focus on temporary nurse staffing therefore risks overshadowing the more fundamental challenges of sustaining a stable and resilient nursing workforce. At the policy level, this focus may reflect a tendency to concentrate debate on a visible and seemingly tractable issue, while deeper and more complex workforce challenges are harder to address and consequently receive less sustained attention.

Addressing these structural issues ultimately depends on the leadership capacity of those managing care delivery at the unit level. Nurse managers are central for patient outcomes, staff retention, and the functioning and transformation of healthcare organizations [29, 30]. They are expected to be visionary, knowledgeable, and effective communicators who act as change agents and provide supportive leadership to their staff [31]. At the same time, they must ensure the smooth functioning of the ward and coordinate limited staffing resources, often in addition to providing direct patient care in some capacity. These overlapping expectations illustrate the scope and complexity of the role. In our sample, the median tenure in the managerial position was five years (IQR 3-10), and about one fifth of managers held a university degree. Effective nursing leadership—especially when it fosters empowerment, communication, and teamwork—is positively associated with nurses’ job satisfaction, engagement, and care quality [32, 33]. Despite the importance of these competencies, many nurse managers receive limited preparation and ongoing training, even though such development is linked to leadership effectiveness and job satisfaction [30, 34]. Given the heavy workloads that nurse managers carry [30], it remains an open question whether they have sufficient time, skills, institutional support, and decision-making power to develop strategic or structural responses to the operational challenges they face.

### 4.1 Limitations

The study has limitations. It is set in psychiatric inpatient care within the Swiss healthcare system, which may limit generalizability to other national contexts or clinical specialties where staffing dynamics differ. While the combination of routine data and survey responses provides complementary insights, integration was descriptive rather than inferential. Because only nine months of data were available, potential seasonal patterns in staffing or care demand could not be examined. The study draws on the perspectives of unit managers, therefore the reasons and barriers perceived by nursing staff may differ. Finally, the study did not assess patient or nurse outcomes directly, limiting conclusions about how mismatches between patient care demand and nurse staffing translate into quality or safety.

## 5 Conclusion

Psychiatric nursing illustrates the broader challenge of aligning dynamic patient demand with staffing systems designed for stability and predictability. Unit managers report limited adaptive strategies, and routine data highlight substantial fluctuations in capacity utilization. While flexible working arrangements are discussed as promising, their practical implementation remains constrained by the operational realities of shift-based care.

Future research should focus on two main areas. First, studies should develop and evaluate approaches that improve timely insight into variations in care demand, including both observed and anticipated peaks and valleys in nurse workload, as such insight is a prerequisite for effective action. Second, research should examine a broader range of responses to patient-side demand fluctuations. While some interventions may target nurse workforce capacity, others may involve adaptations in care delivery, such as the temporary rationing or prioritization of care based on previously agreed-upon organizational consensus. Additional avenues include responses that draw on professionals from other healthcare disciplines and adjustments to their workflows, particularly in settings with strong interprofessional collaboration, including but not limited to psychiatric care. Considering these approaches alongside staffing-related interventions may help identify more strategies to ensure safe, high-quality patient care and protect staff well-being in the context of workforce shortages.

For practice, unit managers should be supported not only with leadership training and decision autonomy but also with tools that enable them to better assess and interpret changes in demand, possibly by utilizing routine data. Integrating such data-driven decision support could help shift workforce management from reactive problem-solving toward proactive planning.

At the organizational level, translating recurring operational challenges into structural solutions requires active administrative leadership and coordination beyond individual units. This includes greater openness to innovation in workforce planning, such as alternative scheduling models, shared staffing resources, and transparent communication about the limits of what organizations can realistically provide under financial and staffing constraints. However, persistent shortages of financial and human resources cannot be resolved at the organizational level alone. Addressing these challenges requires policy action directed at the structural conditions shaping nursing, rather than a focus on highly visible but comparatively narrow issues. Strengthening the nursing workforce will therefore depend on long-term system-level strategies that support good working conditions and continuous professional development.

## Data Availability

The raw data supporting the conclusions of this article will be made available by the authors, without undue reservation.
