# Supplementary material for: Unit managers between fluctuating demand and fixed staffing: a quantitative study in psychiatric nursing
Source: Front Health Serv. 2026 Jan 30;6:1751261. doi: 10.3389/frhs.2026.1751261 (PMC12901418; doi:10.3389/frhs.2026.1751261)
Supplement: Supplementary file 1 [file Datasheet1.pdf]

Supplementary File 1: Unit Manager Survey

Note:
The original survey instrument was developed in German. The English version shown in this supplement is an ad-hoc translation provided for informational purposes only. It has not undergone a formal translation or back-translation process and does not represent a validated English version of the survey.

Question	Statement / sub-question	Response options / notes
Consent	-	Yes / No
**Unit characteristics**		
How many beds does the unit have (at 100% occupancy)?	-	Integer (1–99)
How many additional beds can be used on the unit (e.g., emergency/overflow beds)?	-	Integer (0–99)
How many outpatient/day clinic places are available on the unit?	-	Integer (0–99)
Which percentage best describes, in your estimation, the unit's average bed occupancy over the past 12 months?	-	Slider: 0% - 100%
What is the unit's door status?	-	1 = Open<br>2 = Closed<br>3 = Partly open / partly closed
**Staffing positions**		
Do peer support workers work on the unit?	-	Yes / No
Number of hours per week worked by peer support workers on the unit	-	Number (0–99); shown if peer support workers = Yes
How many nursing positions does the unit have and how many are currently filled?	Registered nurses (planned postions)	Number (1–30); decimals allowed (use dot)
	Registered nurses (filled positions)	Number (1–30); decimals allowed (use dot)
	Licensed practical nurses and care assistants (planned positions)	Number (0–30); decimals allowed (use dot)
	Licensed practical nurses and care assistants (filled positions)	Number (0–30); decimals allowed (use dot)
**Typical staffing**		
What does typical staffing on your unit look like?	Weekdays, registered nurses, early/day shift	Integer; enter NA if the shift type does not exist on your unit. (1–30)
	Weekdays, registered nurses, late shift	Integer; enter NA if the shift type does not exist on your unit. (1–30)

	Weekdays, registered nurses, night shift	Integer; enter NA if the shift type does not exist on your unit. (1–30)
	Weekdays, licensed practical nurses and care assistants, early shift	Integer; enter NA if the shift type does not exist on your unit. (0–30)
	Weekdays, licensed practical nurses and care assistants, late shift	Integer; enter NA if the shift type does not exist on your unit. (0–30)
	Weekdays, licensed practical nurses and care assistants, night shift	Integer; enter NA if the shift type does not exist on your unit. (0–30)
	Weekends/holidays, registered nurses, early/day shift	Integer; enter NA if the shift type does not exist on your unit. (1–30)
	Weekends/holidays, registered nurses, late shift	Integer; enter NA if the shift type does not exist on your unit. (1–30)
	Weekends/holidays, registered nurses, night shift	Integer; enter NA if the shift type does not exist on your unit. (1–30)
	Weekends/holidays, licensed practical nurses and care assistants, early shift	Integer; enter NA if the shift type does not exist on your unit. (0–30)
	Weekends/holidays, licensed practical nurses and care assistants, late shift	Integer; enter NA if the shift type does not exist on your unit. (0–30)
	Weekends/holidays, licensed practical nurses and care assistants, night shift	Integer; enter NA if the shift type does not exist on your unit. (0–30)
**Patient assignment**		
How many patients is a registered nurse typically responsible for?	Weekdays, early/day shift	Integer (1–50)
	Weekdays, late shift	Integer (1–50)
	Weekdays, night shift	Integer (1–50)
	Weekends/holidays, early/day shift	Integer (1–50)
	Weekends/holidays, late shift	Integer (1–50)
	Weekends/holidays, night shift	Integer (1–50)
**Education mix**		
Among the registered nurses on your unit, what proportion do you estimate have a Bachelor’s degree?	-	Percent (0–100)
**Advanced nursing roles**		
Can you draw on cross-unit Advanced Practice Nurses (APN) or Master’s-	-	Yes / No

level nurse experts (e.g., in complex patient situations)?		
Can you draw on cross-unit HöFa II-level nurse experts *(Note: older Swiss qualification, approximately comparable to a Master's level)?*	-	Yes / No
**Management strategies**		
How often do you use the following options to balance fluctuations in staffing or patient workload (too high or too low patient demand)?	We leave staffing levels as planned	1 = Very often<br>2 = Often<br>3 = Sometimes<br>4 = Rarely<br>5 = Very rarely<br>6 = Never
	We reduce staffing levels (employees stay at home)	1 = Very often<br>2 = Often<br>3 = Sometimes<br>4 = Rarely<br>5 = Very rarely<br>6 = Never
	Overtime by employees (staying longer at work)	1 = Very often<br>2 = Often<br>3 = Sometimes<br>4 = Rarely<br>5 = Very rarely<br>6 = Never
	Bringing in employees from leave if necessary	1 = Very often<br>2 = Often<br>3 = Sometimes<br>4 = Rarely<br>5 = Very rarely<br>6 = Never
	Support from colleagues from other units with a lower workload	1 = Very often<br>2 = Often<br>3 = Sometimes<br>4 = Rarely<br>5 = Very rarely<br>6 = Never
	Employees helping out in units with a higher workload	1 = Very often<br>2 = Often<br>3 = Sometimes<br>4 = Rarely<br>5 = Very rarely<br>6 = Never
	On-call duty for our unit	1 = Very often<br>2 = Often<br>3 = Sometimes<br>4 = Rarely<br>5 = Very rarely<br>6 = Never
	Additional employees from temporary agencies	1 = Very often<br>2 = Often<br>3 = Sometimes<br>4 = Rarely

		5 = Very rarely<br>6 = Never
	Additional employees from the float pool	1 = Very often<br>2 = Often<br>3 = Sometimes<br>4 = Rarely<br>5 = Very rarely<br>6 = Never
	Employees with flexible working time accounts do (not) come to work	1 = Very often<br>2 = Often<br>3 = Sometimes<br>4 = Rarely<br>5 = Very rarely<br>6 = Never
	We transfer patients to other units	1 = Very often<br>2 = Often<br>3 = Sometimes<br>4 = Rarely<br>5 = Very rarely<br>6 = Never
	We close beds (staffing is reduced accordingly)	1 = Very often<br>2 = Often<br>3 = Sometimes<br>4 = Rarely<br>5 = Very rarely<br>6 = Never
	Do you use any other options?	Yes / No
	Other: please specify	Free text
	Other (as specified above)	1 = Very often<br>2 = Often<br>3 = Sometimes<br>4 = Rarely<br>5 = Very rarely<br>6 = Never
**Flexible working arrangements**		
How important do you consider flexible working time models for nurses?	-	1 = Very important<br>2 = Important<br>3 = Neither important nor unimportant<br>4 = Unimportant<br>5 = Very unimportant
Which forms of flexible working time models do you offer? (Multiple answers possible)	-	Checkbox (select all that apply):<br>1 = None<br>2 = Flexible working time per month<br>3 = Annualised hours (working time calculated over the year)<br>4 = Flexible additional hours (flexible time accounts)<br>5 = Flextime (flexible

		start/end times per shift)<br>6 = Longer daily working time (e.g., 12-hour shifts) with more days off per week<br>7 = Combination of flexible and fixed employment (fixed minimum with optional additional hours)<br>8 = Float pool across multiple units<br>9 = Other (specify)
	Other: please specify	Free text
In your view, what are the most important reasons for introducing flexible working time models?	None, there are no important reasons for this	1 = Fully applies<br>2 = Mostly applies<br>3 = Rather does not apply<br>4 = Does not apply at all
	Staff motivation	1 = Fully applies<br>2 = Mostly applies<br>3 = Rather does not apply<br>4 = Does not apply at all
	Improving staff work–life balance	1 = Fully applies<br>2 = Mostly applies<br>3 = Rather does not apply<br>4 = Does not apply at all
	Retention of staff	1 = Fully applies<br>2 = Mostly applies<br>3 = Rather does not apply<br>4 = Does not apply at all
	Better ability to respond to workload fluctuations	1 = Fully applies<br>2 = Mostly applies<br>3 = Rather does not apply<br>4 = Does not apply at all
	Other (please specify below)	1 = Fully applies<br>2 = Mostly applies<br>3 = Rather does not apply<br>4 = Does not apply at all
	Other: please specify	Free text (associated with ‘Other’ item above)
	-	Yes / No
Flexible working time models: In your view, how high is your nurses’ interest in flexible working time models?	-	Slider: 1 (very low) to 10 (very high)
Flexible working time models: Where do you see barriers to introducing flexible working time models?	Concerns about maintaining shift operations	1 = Fully applies<br>2 = Mostly applies<br>3 = Rather does not apply<br>4 = Does not apply at all
	Complexity of duty scheduling	1 = Fully applies<br>2 = Mostly applies<br>3 = Rather does not apply<br>4 = Does not apply at all

	Concerns about continuity of care	1 = Fully applies<br>2 = Mostly applies<br>3 = Rather does not apply<br>4 = Does not apply at all
	Concerns about opportunities for professional exchange	1 = Fully applies<br>2 = Mostly applies<br>3 = Rather does not apply<br>4 = Does not apply at all
	Barriers related to employment law	1 = Fully applies<br>2 = Mostly applies<br>3 = Rather does not apply<br>4 = Does not apply at all
	Lack of support from the HR department	1 = Fully applies<br>2 = Mostly applies<br>3 = Rather does not apply<br>4 = Does not apply at all
	Lack of support from company management	1 = Fully applies<br>2 = Mostly applies<br>3 = Rather does not apply<br>4 = Does not apply at all
	Lack of relevance of the topic	1 = Fully applies<br>2 = Mostly applies<br>3 = Rather does not apply<br>4 = Does not apply at all
	Are there any other barriers?	Yes / No
	Other: please specify	Free text
	Other	1 = Fully applies<br>2 = Mostly applies<br>3 = Rather does not apply<br>4 = Does not apply at all
**High workload**		
How often are beds closed on your unit (e.g., due to staff shortages)?	-	1 = Never<br>2 = 1-4 times per year<br>3 = 5-10 times per year<br>4 = Once per month<br>5 = Several times per month<br>6 = Several times per week
When situations of high workload for nurses occur on the unit, they are most likely caused by…	A high number of patients	Scale 1–10 (1 = Does not apply at all; 10 = Applies fully)
	The high level of care required by individual or multiple patients	Scale 1–10 (1 = Does not apply at all; 10 = Applies fully)
	Too few nursing staff	Scale 1–10 (1 = Does not apply at all; 10 = Applies fully)
	Too few experienced nursing staff	Scale 1–10 (1 = Does not apply at all; 10 = Applies fully)

	Problems within the interdisciplinary team	Scale 1–10 (1 = Does not apply at all; 10 = Applies fully)
	High administrative workload	Scale 1–10 (1 = Does not apply at all; 10 = Applies fully)
	Are there any other causes?	Yes / No
	Other: please specify	Free text
	Other	Scale 1–10 (1 = Does not apply at all; 10 = Applies fully)
**Temporary staff**		
How often are agency/temporary staff used on your unit?	-	1 = Never<br>2 = 1-4 times per year<br>3 = 5-10 times per year<br>4 = Once per month<br>5 = Several times per month<br>6 = Several times per week
On your unit, has the use of agency/temporary staff since 2019…	-	1 = Increased<br>2 = Decreased<br>3 = Not changed
**Respondent information**		
What is your role/function?	-	1 = Unit manager<br>2 = Deputy unit manager<br>3 = Unit manager of multiple units<br>4 = Deputy unit manager of multiple units<br>5 = Other (specify)
	Other: please specify	Free text
Which qualification have you completed?	-	1 = Nurse with university degree (Bachelor or Master)<br>2 = Nurse with higher vocational education<br>3 = Other (specify)
	Other: please specify	Free text
How many years have you worked in this hospital?	-	Integer (1–50); rounded up to a whole number
How many of those years have you been in a management role?	-	Integer (1–50); rounded up to a whole number
How many years have you worked in nursing in total?	-	Integer (1–50); rounded up to a whole number
